# Flavonoids and 5-Aminosalicylic Acid Inhibit the Formation of Neutrophil Extracellular Traps

**DOI:** 10.1155/2013/710239

**Published:** 2013-12-07

**Authors:** Tina Kirchner, Eva Hermann, Sonja Möller, Matthias Klinger, Werner Solbach, Tamás Laskay, Martina Behnen

**Affiliations:** ^1^Institute for Medical Microbiology and Hygiene, University of Lübeck, Ratzeburger Allee 160, 23538 Lübeck, Germany; ^2^Institute of Anatomy, University of Lübeck, Ratzeburger Allee 160, 23538 Lübeck, Germany

## Abstract

Neutrophil extracellular traps (NETs) have been suggested to play a pathophysiological role in several autoimmune diseases. Since NET-formation in response to several biological and chemical stimuli is mostly ROS dependent, in theory any substance that inhibits or scavenges ROS could prevent ROS-dependent NET release. Therefore, in the present comprehensive study, several antioxidative substances were assessed for their capacity to inhibit NET formation of primary human neutrophils *in vitro*. We could show that the flavonoids (−)-epicatechin, (+)-catechin hydrate, and rutin trihydrate as well as vitamin C and the pharmacological substances *N*-acetyl-L-cysteine and 5-aminosalicylic acid inhibited PMA induced ROS production and NET formation. Therefore, a broad spectrum of antioxidative substances that reduce ROS production of primary human neutrophils also inhibits ROS-dependent NET formation. It is tempting to speculate that such antioxidants can have beneficial therapeutic effects in diseases associated with ROS-dependent NET formation.

## 1. Introduction

Neutrophils are essential effector cells of the innate antimicrobial defense. As professional phagocytes neutrophils ingest and kill invading microorganisms. However, via the release of antimicrobial peptides and reactive oxygen species (ROS), they are also able to kill pathogens independently of their phagocytic function [1]. In addition, neutrophils can capture and kill pathogens by releasing neutrophil extracellular traps (NETs) [2]. NETs are complex three-dimensional structures containing several antimicrobial neutrophil granule proteins attached to the DNA backbone [2]. They are mostly released from activated neutrophils that undergo NETosis, a form of cell death differing from apoptosis and necrosis [3]. This programmed, lytic cell death is mediated by ROS, such as superoxide (O_2_^∙−^) and hypochlorite (OCl^−^) produced by the enzymes NADPH oxidase and myeloperoxidase (MPO) [3–10]. Although ROS production and activity of NADPH oxidase and MPO have been claimed as being essential in the formation of NETs in response to several biological and chemical stimuli, it has also been reported that some microorganisms (*S. aureus*, *L. donovani*) and certain stimuli (MIP-2) are able to induce NETs in a ROS independent manner [11–13].

Several studies suggest a pathophysiological role of NETs and NET components in autoimmune diseases such as small-vessel vasculitis, lupus nephritis, systemic lupus erythematosus (SLE), psoriasis, and rheumatoid arthritis [2, 14]. Consequently, inhibition of NET release could result in beneficial therapeutic effects in these diseases. Since NET release is mostly dependent on ROS and NADPH oxidase- and MPO activity generated ROS [4, 9, 15], also in autoimmune inflammatory diseases such as rheumatoid arthritis [16] or SLE [17], any molecule that inhibits the generation of ROS or scavenges ROS should be able to prevent the ROS-dependent NETosis. As it is known that inhibitors of NADPH oxidase and MPO, such as diphenyleneiodonium chloride (DPI) or sodium azide, have unspecific or toxic effects on host cells [18], molecules lacking such toxic effects could be candidates for medical application. Among these are radical scavengers and pharmacological drugs. Only few studies have addressed the effect of these substances on the NET release of human neutrophils [9, 19–22]. In the present comprehensive study, a wide range of substances with antioxidative activity were assessed for their capacity to inhibit ROS-dependent NET formation of primary human neutrophils *in vitro* to identify molecules with the potential to be therapeutic agents in NET-related diseases. NET production of freshly isolated neutrophils was induced by phorbol myristate acetate (PMA), the best characterized ROS-dependent NETosis model. The antioxidants used in this study can be divided into the three major groups: flavonoids, vitamins, and other pharmacological substances. The antioxidative effect of these substances is related not only to their ability to scavenge ROS like superoxide (O_2_^∙−^), hydroxyl radicals (OH^∙^) or hypochlorite (OCl^−^). In addition to their scavenging effect flavonoids and anti-inflammatory drugs such as 5-aminosalicylic acid and acetylsalicylic acid can inhibit the MPO, as well [23–26]. From the group of flavonoids, (+)-catechin hydrate, (–)-epicatechin, and rutin trihydrate were used. Two vitamins with well-known antioxidant activities, vitamin C (ascorbic acid) and E ((*α*)-tocopherol), were also tested in our study. Furthermore, *N*-acetyl-L-cysteine, the precursor of the natural antioxidant glutathione [27], and the hormone of the pineal gland with ROS scavenger activity melatonin [28] were tested.

The present study reveals for the first time an inhibitory effect of the flavonoids epicatechin, catechin hydrate, and rutin trihydrate as well as of the pharmacological drug 5-aminosalicylic acid on the ROS dependent NET formation. The same inhibitory effect on ROS and NET formation was observed for vitamin C as well as for the drug *N*-acetyl-L-cysteine. All these substances showed a significant antioxidative activity on the formation of ROS and inhibited the release of ROS-dependent NETs from PMA stimulated human neutrophils *in vitro*, while other effector functions such as phagocytosis, chemotaxis, and degranulation were not affected. Acetylsalicylic acid, *α*-tocopherol, and melatonin did not have an effect on the NET formation in the present study.

## 2. Materials and Methods

### 2.1. Ethics Statement

The blood collection was conducted with the understanding and the consent of each participant and approved by the ethical committee of the Medical Faculty of the University of Lübeck (05-124).

### 2.2. Isolation and Culture of Primary Human Neutrophils

Peripheral blood was collected by venipuncture from healthy adult volunteers using lithium-heparin. Neutrophils were isolated via density gradient centrifugation as described [29]. The cell preparations contained >99.9% granulocytes as determined by morphological examination of Giemsa-stained cytocentrifuged slides (Shandon, Pittsburgh, PA, USA). Neutrophils were cultured in complete medium (RPMI 1640 medium supplemented with 50 *μ*M 2-mercaptoethanol, 10 mM HEPES, 10% heat inactivated fetal bovine serum (all from Sigma-Aldrich, Steinheim, Germany)) containing 4 mM L-glutamine, 100 U/mL penicillin, 100 *μ*g/mL streptomycin (all from Biochrom, Berlin, Germany) at 37°C in a humidified air atmosphere containing 5% CO_2_.

### 2.3. Antioxidants

Antioxidants are scavengers of various reactive oxygen species (ROS) and were used in the present study to evaluate their impact on the release of ROS as well as NET formation on PMA-(Sigma-Aldrich, Steinheim, Germany) stimulated neutrophils. In all experiments the neutrophils were preincubated with antioxidants for 30 min at 37°C prior to the NET induction by PMA. Unstimulated neutrophils treated with antioxidants served as control to exclude nonspecific effects of the antioxidants in the performed ROS- and NET-assays. As antioxidants the vitamin C L-ascorbic acid (0.2–2 mM) and the vitamin E (±)-*α*-tocopherol (50 *μ*M, *α*-tocopherol, both from Sigma-Aldrich) as well as the flavonoids (−)-epicatechin (epicatechin, 4–100 *μ*M), (+)-catechin hydrate (catechin hydrate, 4–100 *μ*M, both from Sigma-Aldrich), and rutin trihydrate (0.1–150 *μ*M, Carl Roth, Karlsruhe, Germany) were used. In addition, the antioxidative effect of melatonin (1-2 mM, Sigma-Aldrich), 5-aminosalicylic acid (0.005, 0.25, 0.5 mM, 5-ASA, TCI Europe N.V., Eschborn, Germany), acetylsalicylic acid (1 mM, ASS), and *N*-acetyl-L-cysteine (5–10 mM, NAC, all from Sigma-Aldrich) was investigated. Urea (1 mM) which has no scavenging capacity and no influence on the ROS-mediated signal pathways was used as a negative control. As additional control, neutrophils were incubated in medium alone and, if necessary, in medium containing the relevant solvent. DMSO and ethanol (EtOH) represent the solvent controls for the flavonoids, acetylsalicylic acid, *α*-tocopherol, and melatonin. All antioxidants were prepared freshly for each experiment and sterile filtered. The whole preparations for the experiments were done under sterile conditions and in the dark.

### 2.4. Detection of Reactive Oxygen Species (ROS) Release

4 × 10^5^ freshly isolated human neutrophils (2 × 10^6^/mL) were seeded in a custom-made modified RPMI-1640 medium without phenol red and sodium hydrogen carbonate containing 20 mM HEPES (Biochrom, Berlin, Germany) in a 96 nunclon delta white microwell plate (Nunc, Langensbold, Germany) and preincubated with or without antioxidants for 30 min at 37°C and 5% CO_2_. Two assays were performed to detect the ROS production.

#### 2.4.1. Luminol Assay

The luminol-amplified chemiluminescence assay was used to detect the sum of intra- and extracellular ROS. Luminol (5-amino-2,3-dihydro-1,4-phthalazindione) is excited by MPO-derived metabolites [30]. During their activation, neutrophils degranulate and release MPO from azurophil granules. Due to these effects, both intracellular and extracellular ROS are detected by this technique.

Neutrophils pre-incubated with or without antioxidants were stained with 0.06 mM luminol (Sigma-Aldrich) and stimulated with 20 nM PMA or left unstimulated. Immediately after the stimulation, the increase of the chemiluminescence resulting from the ROS production was continuously analyzed over 1 h at 37°C by an Infinite 200 PRO reader and the Tecan i-control 1.8 software (Tecan, Crailsheim, Germany). Samples without PMA treatment (medium) as well as neutrophils pre-incubated with solvent control were used as control. DMSO and EtOH were used as solvent controls for the particular antioxidants dissolved in this medium.

#### 2.4.2. Lucigenin Assay

Lucigenin is specifically reduced by superoxide anion radicals and releases energy in form of light as a consequence. MPO-derived ROS do not exite lucigenin [30, 31]. Therefore, the lucigenin-enhanced assay was used to study the superoxide production of neutrophils. Because of its size, lucigenin cannot penetrate the cell membrane and detects only extracellular but not intracellular ROS [32]. Neutrophils were treated as described previously but instead of 0.06 mM luminol, 0.2 mM lucigenin (Alexis, Loerrach, Germany) was added.

### 2.5. Induction and Detection of Neutrophil Extracellular Traps

Staining and detection of extracellular DNA with the non cell-permeable DNA dye SYTOXgreen is a commonly used and well established method to study the formation of NETs [4, 33–35]. To assess ROS-dependent NET formation by PMA-stimulated neutrophils pre-incubated with or without antioxidants 2 × 10^5^ freshly isolated human neutrophils (1 × 10^6^/mL) in NET medium (a custom-made modified RPMI-1640 medium without phenolred and sodium hydrogen carbonate containing 20 mM HEPES (Biochrom, Berlin, Germany), supplemented with 0.5% human serum albumin (Behring, Marburg, Germany) and 10 mM HEPES buffer (PAA, Pasching, Austria)) were seeded to a 96 nunclon delta black microwell plate (Nunc) and pre-incubated with or without antioxidants for 30 min at 37°C, 5% CO_2_. Afterwards, 5 *μ*M SYTOXgreen (Life technologies, Darmstadt, Germany) was added, followed by stimulation with 20 nM PMA (Sigma-Aldrich). To exclude a non-specific effect, for example autofluorescence of antioxidants in the NET-assay, unstimulated neutrophils treated with antioxidants served as control. The fluorescence of NET-bound SYTOXgreen (excitation: 488 nm, emission: 510 nm) was analyzed over a period of 5 h every 5 min at 37°C by the infinite 200 reader and the Tecan i-control 1.8 software (Tecan). Medium and solvents (DMSO, EtOH) were used as controls.

### 2.6. Visualization of NETs by Fluorescence and Scanning Electron Microscopy

Fluorescence microscopy (FM) and scanning electron microscopy (SEM) were performed to visualize NET formation and to confirm the results of the SYTOXgreen assay. Freshly isolated human neutrophils (1 × 10^6^/mL) in NET medium were preincubated with antioxidants or medium/solvent control for 30 min at 37°C. 3 × 10^5^ neutrophils were seeded either on a black 96 well *μ*-plate (Ibidi, Planegg/ Martinsried, Germany) for the fluorescence microscopy or on a 8 well *μ*-slide (Ibidi) for immunohistochemical staining. After preincubation, the samples were stained with 100 nM SYTOXgreen (Life technologies). NET formation was induced by 20 nM PMA (Sigma-Aldrich) for 3 h. Samples without PMA were used as control. Afterwards the samples were fixed with 4% paraformaldehyde (Sigma) for 10 min at room temperature. The supernatant was carefully removed. After washing with nuclease-free water (Sigma-Aldrich), the samples were fixed with mounting medium (Ibidi) and analyzed with the fluorescence microscope BZ9000E using the BZ II Analyzer software (both from KEYENCE, Neu-Isenburg, Germany). For intra- and extracellular immunohistochemical staining of neutrophil myeloperoxidase, the samples were fixed with 4% paraformaldehyde (Sigma) for 10 min, permeabilized with a 0.5% solution of Triton-X (Merck, Darmstadt, Germany) for 1 min, and washed three times with PBS (PromoCell, Heidelberg, Germany). Unspecific binding sites were blocked by a buffer containing normal goat serum (Jackson ImmunoResearch Europe, Newmarket, UK) in PBS in a ratio of 1 : 20 for 30 min at 37°C. Afterwards, the samples were incubated with the primary antibody mouse anti-human myeloperoxidase (AbD Serotec, Düsseldorf, Germany, 1 : 500) for 1 h at 37°C. Following washing with PBS three times, the secondary antibody goat anti-mouse (Cy3-conjugated AffiniPure *F*(*ab*′)_2_ fragment goat anti-mouse IgG (H+L), Jackson ImmunoResearch Europe) was added (1 : 500) for 1 h at 37°C. The samples were washed three times with PBS and stained with 100 nM SYTOXgreen (Life technologies) for 10 min in the dark. Finally, samples were washed three times with nuclease-free water (Sigma-Aldrich) and fixed using the mounting medium (ibidi).

The scanning electron microscopy was done with 1 × 10^6^ neutrophils per sample settled on thermanox coverslips (Nunc) in a 24 well culture plate (Greiner-Bio-One). NETosis was induced by 20 nM PMA (Sigma-Aldrich) after preincubation with antioxidants. After incubation for 3.5 h at 37°C, the supernatant was carefully removed and samples were fixed with Monti-Graziadei solution (2% glutaraldehyde, 0.6% paraformaldehyde in 0.1 M cacodylate buffer, bpH 7.2) for 2 days. The samples were dehydrated in a rising alcohol series (30, 40, 50, 60, 70, 80, 90, and 100% for 15 min each), placed on aluminium slides, sputtered with gold or platin, and were examined in a SEM 505 (Philips, Eindhoven, Holland).

### 2.7. Statistical Analysis

**Figure 1:**
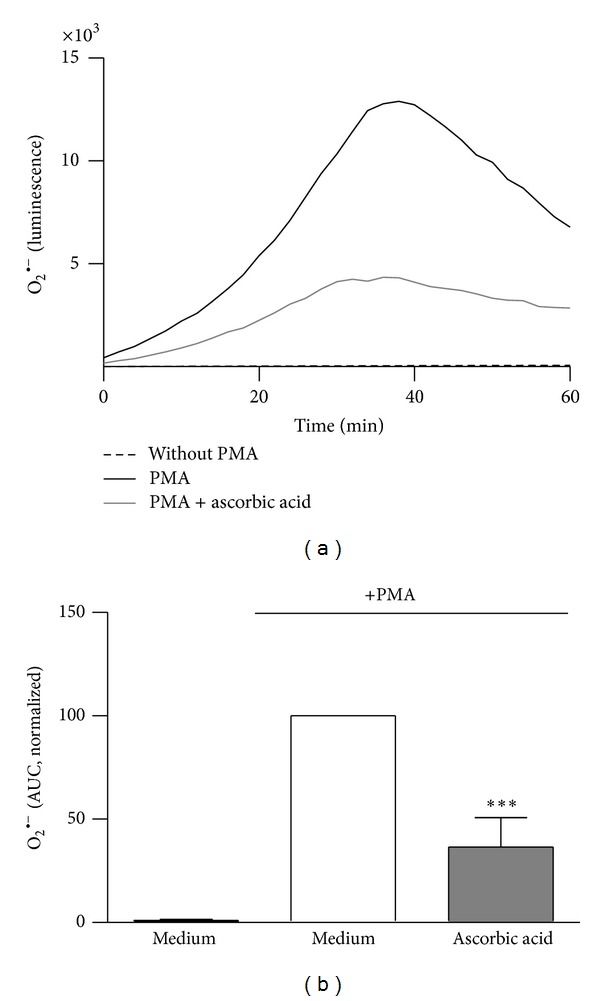
Analysis of the ROS production in neutrophils by calculation of the area under the curve. The extracellular ROS formation of neutrophils incubated with 2 mM ascorbic acid decreases as measured by the lucigenin amplified chemiluminescence assay. Neutrophils were coincubated with or without ascorbic acid for 30 min at 37°C and the ROS release was induced by PMA. The area under the curve (AUC) of the time kinetics (1 h) of superoxide (O_2_^∙−^) release of each curve (a) was calculated and represented as bar graph. Data show mean ± SD from 3 independent experiments of 3 different donors; ***P < 0.001 (b). The figure is representative for the analysis of all experiments detecting the ROS and NET release.

The kinetics of the experiments detecting the ROS and NET release were analyzed by calculating the area under the curve (AUC) of each sample (Figure 1(a)). These AUCs were represented as bar graphs (Figure 1(b)). Statistical analysis was performed with the GraphPad Prism software 5 using the one-way ANOVA test and Bonferroni posttest. Because of donor-dependent differences, all data were normalized against neutrophils stimulated with PMA (Medium). For statistical analysis, either medium or solvent control (DMSO, EtOH) was used as reference.

## 3. Results

### 3.1. The Antioxidants Used in the Present Study Do Not Induce Apoptosis or Necrosis in Human Neutrophils *In Vitro*

The concentrations of antioxidants were chosen either based on the literature [22, 23, 36–41] or on our own preliminary studies. To exclude toxic or apoptosis-inducing effects, freshly isolated human neutrophils were incubated with the antioxidants over 5 h and double-stained with annexin V-FITC and propidium iodide. Annexin V-FITC binds on the exposed phosphatidylserine of apoptotic neutrophils whereas propidium iodide labels necrotic cells [42]. None of the applied antioxidants exerted a toxic or apoptosis-inducing effect after 5 h as compared to the appropriate solvent control see Supplementary Material available online at http://dx.doi.org/10.1155/2013/710239.

### 3.2. Flavonoids, Vitamin C, 5-ASA and NAC Inhibit the PMA Stimulated ROS Release of Human Neutrophils

Intracellular and/or extracellular ROS were analyzed in cultures of PMA stimulated neutrophils preincubated with antioxidants by using two ROS detection methods. MPO-derived ROS such as hypochlorous acid have been shown to be involved in the induction of NET release [4, 9]. Thus, as a first approach, the luminol-amplified chemiluminescence assay was applied to assess the effect of antioxidants on the intra- and extracellular ROS production. In this assay hydrogen peroxide and MPO-derived ROS, such as hypochlorite and hydroxyl radicals [30, 31], oxidize luminol, which releases light detectable as chemiluminescence.

**Figure 2:**
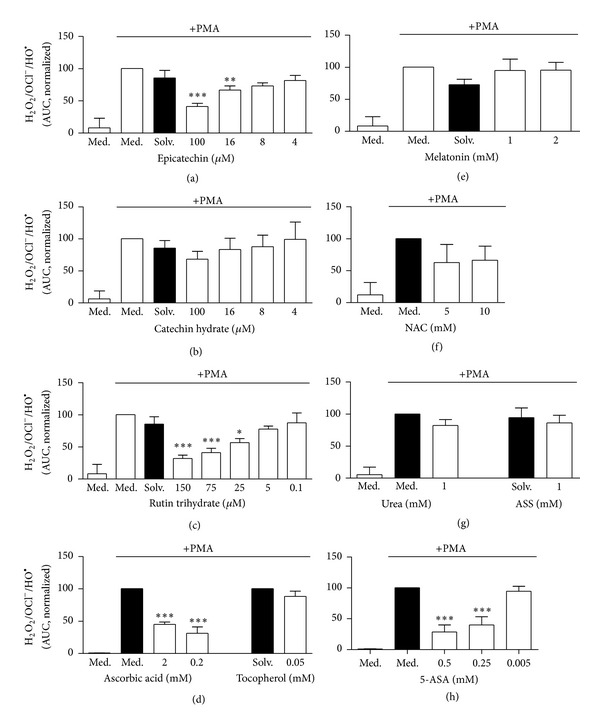
Influence of antioxidants on neutrophil ROS formation as measured by the luminol assay. Freshly isolated neutrophils were preincubated with or without antioxidants for 30 min at 37°C. Following addition of luminol (0.06 mM) and induction by 20 nM PMA, the kinetic of ROS formation was measured over a period of 1 h at 37°C. ROS formation of (a) epicatechin, (b) catechin hydrate, (c) rutin trihydrate, (d) ascorbic acid and tocopherol, (e) melatonin, (f) *N*-acetyl-L-cysteine (NAC), (g) urea and acetylsalicylic acid (ASS), and (h) 5-aminosalicylic acid (5-ASA) was quantified by calculation of the area under the curve (AUC). Data were normalized to the sample with PMA-stimulated neutrophils without antioxidants (Med.). Data show mean ± SD from 3 ((d), (f), (g) (urea), (h)), 5 ((a), (c), (e), (g) (ASS)) or 7 (b) independent experiments with 2 ((g), ASS), 3 ((a), (d), (e), (f), (g) (urea)), 4 ((b), (c)) and 5 (h) different donors. ***P < 0.001; **P < 0.01; *P < 0.05 as compared to the PMA-stimulated medium or solvent control (Med., Solv., black column).

We observed that the flavonoids epicatechin and rutin trihydrate as well as ascorbic acid and 5-aminosalicylic acid (5-ASA) strongly and significantly inhibited neutrophil ROS production in a concentration-dependent manner (Figures 2(a), 2(c), 2(d), and 2(h)), indicating that these antioxidants scavenge/affect MPO-derived ROS. After exposure to *N*-acetyl cysteine (NAC) a tendency of lower ROS production was observed. However, the differences were statistically not significant (Figure 2(f)). No major inhibitory effect on the ROS was observed after exposure of neutrophils to catechin hydrate, tocopherol, melatonin, ASS, and urea control (Figures 2(b), 2(d), 2(e), and 2(g)).

**Figure 3:**
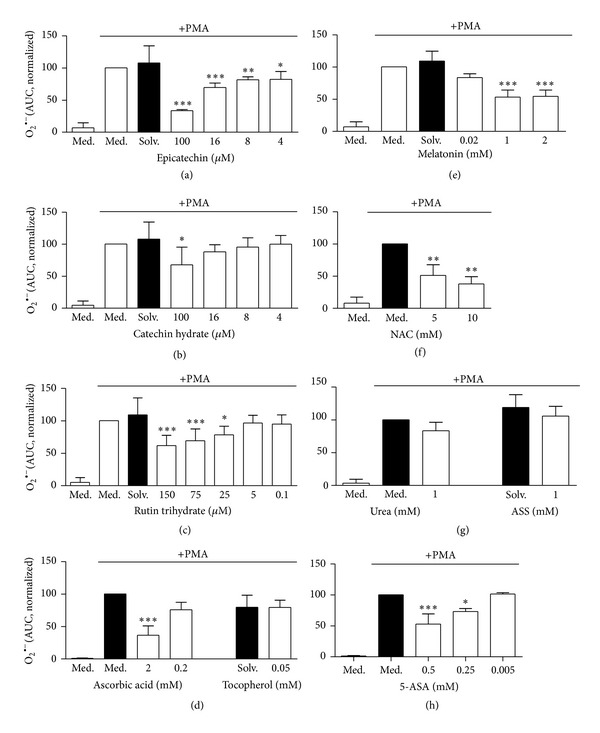
Influence of antioxidants on extracellular ROS formation of neutrophils as measured by the lucigenin assay. Neutrophils were preincubated with or without antioxidants for 30 min at 37°C. Following addition of lucigenin (0.2 mM), ROS formation was induced by 20 nM PMA and the kinetic of O_2_^∙−^-formation was measured over a period of 1 h at 37°C. O_2_^∙−^-formation of (a) epicatechin, (b) catechin hydrate, (c) rutin trihydrate, (d) ascorbic acid and tocopherol, (e) melatonin, (f) *N*-acetyl-L-cytseine (NAC), (g) urea and actetylsalicylic acid (ASS), and (h) 5-aminosalicylic acid (5-ASA) was quantified by calculation of the area under the curve (AUC). Data were normalized to the sample of PMA-stimulated neutrophils without antioxidants (Med.). Data show mean ± SD from 3 ((d), (f), (g) (urea)), 5 ((a), (e), (g) (ASS), (h)), 6 (c) or 7 (b) independent experiments with 2 ((g), ASS), 3 ( (a), (d), (e), (f), (g) (urea)), 4 (b) and 5 ((c), (h)) different donors. ***P < 0.001; **P < 0.01; *P < 0.05 as compared to the PMA-stimulated medium or solvent control (Med., Solv., black column).

As a second approach, the effect of various antioxidants on the ROS production was investigated by using the lucigenin-amplified chemiluminescence assay which detects extracellular ROS, mainly superoxide anions [30, 31]. Extracellular superoxide spontaneously dismutates to hydrogen peroxide, which has been shown to induce NETs when added extracellularly [3]. Moreover, extracellular MPO released upon activation/degranulation and/or NETosis converts hydrogen peroxide to hypochlorite, an inducer of NETosis [9]. The interaction of hypochlorite with hydrogen peroxide leads to generation of singlet oxygen, which has been reported to be essential for NET formation [15]. In line with the results obtained from the luminol-amplified chemiluminescence assay, a concentration-dependent significant inhibitory effect of both flavonoids epicatechin and rutin trihydrate and of ascorbic acid and 5-ASA on the ROS production was observed (Figures 3(a), 3(c), 3(d), and 3(h)). These data thus show that these antioxidants not only affect MPO-derived ROS such as hypochlorite and hydroxyl radicals but also superoxide. Exposure to pharmacologically relevant concentrations of NAC also resulted in a strong and significant reduction of neutrophil ROS production (Figure 3(f)). The flavonoid catechin hydrate had an inhibitory effect only if used at a concentration of 100 *μ*M (Figure 3(b)). In contrast to the results obtained with the luminol assay, melatonin also significantly reduced the ROS production in a dose-dependent manner, when measured by using the lucigenin assay (Figure 3(e)). These results indicate that melatonin only affects extracellular superoxide, but not intra- and extracellular MPO-derived ROS. No inhibitory effect on the ROS production was observed after exposure of neutrophils to tocopherol, ASS, and urea control (Figures 3(d) and 3(g)). This is in line with the results obtained by using the luminol assay and indicates that these substances have no antioxidative effects on superoxide or on MPO-derived ROS in our experimental settings.

In both ROS-detection assays, no chemiluminescence signal was detected from unstimulated neutrophils treated with the antioxidants or with the relevant solvent control, indicating that the tested substances have no unspecific effects in the used assay system (data not shown).

### 3.3. Flavonoids, Vitamin C, 5-ASA and NAC Inhibit the NET Release by PMA Stimulated Human Neutrophils

Since chemical and biological stimulation of neutrophils mostly induces NETosis in a ROS-dependent manner [4, 6, 9, 15], antioxidants that decrease ROS production should also reduce the ROS-dependent NET formation. Only those concentrations of the antioxidants which had a significant inhibitory effect on intra- and/or extracellular ROS were used to assess the effect on NET release. Antioxidants which had no effect on the ROS release were assessed in the highest concentration which was tested for their effect on the NET release. Human neutrophils were preincubated for 30 min with the indicated substances prior to induction of NET formation by 20 nM PMA, the best characterized inducer of ROS-dependent NETosis. NET release was then quantified by measuring the SYTOXgreen fluorescence over a period of 5 h.

**Figure 4:**
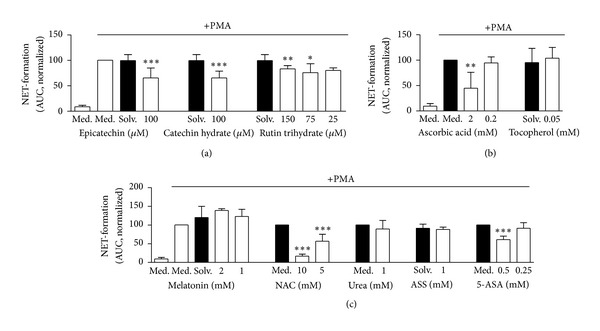
Influence of antioxidants on NET formation as measured by SYTOXgreen fluorescence. Neutrophils were preincubated with or without antioxidants for 30 min at 37°C. In the presence of 5 *μ*M SYTOXgreen, NET formation was induced by 20 nM PMA and assessed for 5 h at 37°C. The NET formation of (a) the flavonoids epicatechin, catechin hydrate and rutin trihydrate, (b) the vitamins ascorbic acid, tocopherol, and (c) other pharmaceutical substances melatonin, *N*-acetyl-L-cytseine (NAC), urea, actetylsalicylic acid (ASS), 5-aminosalicylic acid (5-ASA) was quantified by calculation of the area under the curve (AUC). Data were normalized to the sample of PMA-stimulated neutrophils without antioxidants (Med.). Data show mean ± SD from 4 ((c), melatonin), 5 ((b), (c)), 6 ((a), epicatechin, rutin trihydrate), or 8 ((a), catechin hydrate), independent experiments with 2 ((c), ASS, melatonin), 3 ((c), NAC) 4 ((a), epicatechin, rutin trihydrate), 5 ((b), (c) (urea, 5-ASA)) and 6 ((a), catechin hydrate) different donors. ***P < 0.001; **P < 0.01; *P < 0.05 as compared to the PMA stimulated medium or solvent control (Med., Solv., black column).

As no fluorescence signal was detectable from unstimulated neutrophils treated with antioxidants or solvent controls, we could exclude an autofluorescence or necrosis or NET-inducing effect of all tested substances (data not shown). Pretreatment of neutrophils with the flavonoids epicatechin, catechin hydrate, and rutin trihydrate prior to stimulation with PMA significantly reduced the NET release (Figure 4(a)). The same inhibitory effect on NET formation was observed for ascorbic acid, NAC, and 5-ASA (Figures 4(b) + 4(c)). Therefore, with the exception of melatonin, all substances that significantly reduced PMA-induced ROS production also inhibited PMA-induced NET release (Figures 4(a)–4(c)). All substances that did not display a significant effect on the ROS production, such as tocopherol, urea, and ASS, had no inhibitory effect on the NET release (Figures 4(b) and 4(c)).

**Figure 5:**
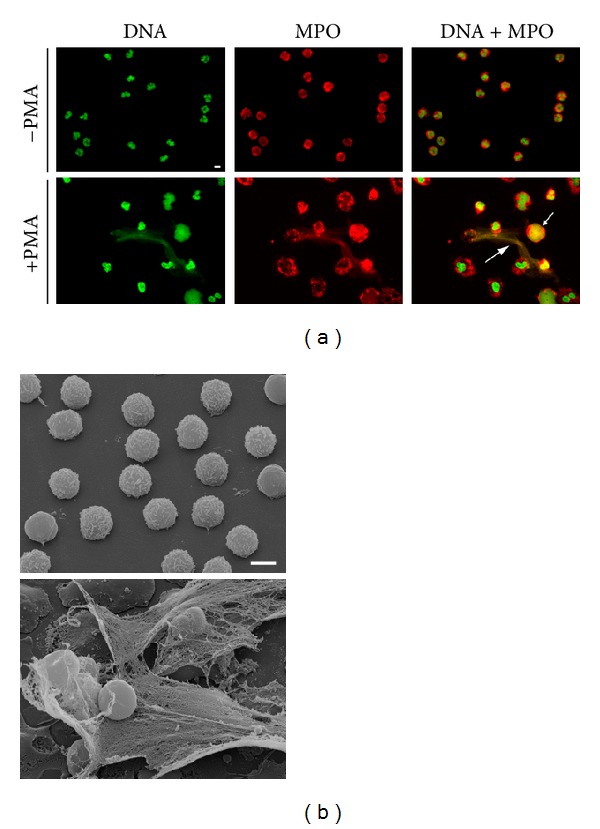
NET formation after PMA stimulation visualized by fluorescence (a) and scanning electron microscopy (b). Upper row shows unstimulated neutrophils; the lower row shows PMA stimulated neutrophils releasing NETs. The scale bar represents 10 *μ*m (fluorescence microscopy) and 5 *μ*m (scanning electron microscopy). (a) Immunohistochemical staining of neutrophils. Green: DNA staining with SYTOXgreen; red: MPO; overlay: SYTOXgreen and MPO. NET release of freshly isolated neutrophils was induced by 10 nM PMA and the samples were fixed after 3 h. Arrows indicate NET fibres and NETotic cells, defined as NETs. (b) Scanning electron microscopy of NET formation induced by 20 nM PMA and fixed after 3.5 h.

To strengthen the results observed by using the quantitative NET-assay, FM and SEM were performed. NET release from untreated or antioxidant-treated neutrophils was visualized 3-4 h after PMA stimulation. Unstimulated neutrophils that do not undergo NETosis show a clear separation between nucleus/chromatin and granula/MPO (Figure 5). Upon PMA-stimulation neutrophils become activated, flattened, and release NETs (Figure 5). NETs appear as fibrous, complex three-dimensional structures (Figure 5(a) large arrow and Figure 5(b)) or have a cloud-like appearance that is several fold bigger than the volume of the viable cells they originate from (Figure 5(a), small arrow) [2]. By immunohistochemical staining for MPO and DNA, we could confirm that NETs are formed by decondensed chromatin structures containing antimicrobial granular proteins (Figure 5(a)). Moreover, neutrophils that undergo NETosis are quite different from viable, apoptotic, or necrotic neutrophils and characterized by a bigger size and intermixing of cellular components (nucleus, granula) (Figure 5(a), small arrow) [2].

**Figure 6:**
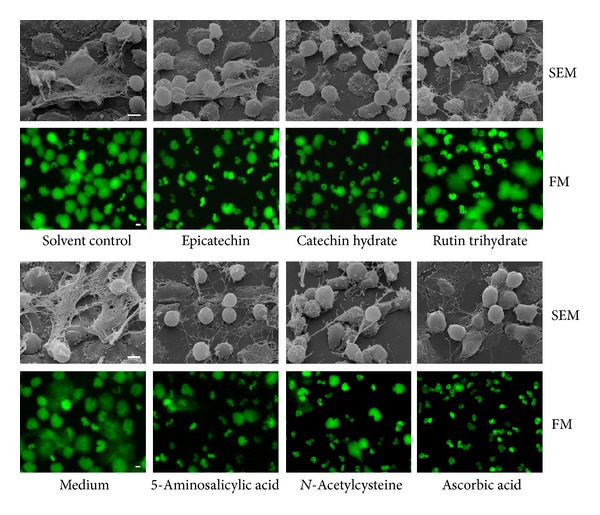
Influence of antioxidants on the formation of NETs as assessed by fluorescence and scanning electron microscopy. Freshly isolated neutrophils were pre-incubated with or without antioxidants (0.5 mM 5-aminosalicylic acid, 10 mM *N*-acetylcysteine, 2 mM ascorbic acid, 0.1 mM epicatechin, 0.1 mM catechin hydrate, 0.075 mM rutin trihydrate) for 30 min at 37°C. The DNA was stained with 100 nM SYTOXgreen for detection with fluorescence microscopy (FM). Afterwards, NET formation was induced by stimulation with PMA (20 nM scanning electron microscopy (SEM), 10 nM FM). The samples were fixed after 4 h (SEM) and 3 h (FM). Scale bars represent 5 *μ*m. Neutrophils stimulated with PMA (Medium) and coincubated with DMSO (solvent control) were used as controls.

The microscopic analysis confirms an inhibitory effect of the flavonoids epicatechin, catechin hydrate, and rutin hydrate on NET release, respectively (Figure 6). Less NETs which are more fragile, but more intact neutrophils, were observed in PMA-stimulated cultures exposed to epicatechin, catechin hydrate or rutin trihydrate compared to the solvent control with wide, compact NETs (Figure 6). 5-ASA, NAC, and ascorbic acid also show a strong inhibitory effect on the NET formation. In comparison to the medium control, almost no NETs are released from 5-ASA-, NAC-, or ascorbic acid-treated cells (Figure 6). Unstimulated neutrophils treated with antioxidants did not undergo NETosis, or necrosis and showed a clear separation between lobulated intact nucleus and granula proteins (data not shown).

Taken together, we could show that all antioxidants that decreased the PMA-induced ROS release of neutrophils were able to inhibit the NET release, with the exception of melatonin.

### 3.4. Antioxidants Used in This Study Do Not Inhibit Degranulation, Chemotaxis and Phagocytic Capacity of Human Neutrophils

In addition to playing a role in NETosis, ROS are involved in other neutrophil functions as well as in signal transduction [43, 44]. By blocking the NADPH oxidase, the main ROS producing enzyme in neutrophils, neutrophils not only fail to produce ROS and NETs [4] but also show an impaired polarization, chemotaxis, adhesion, and phagocytosis [44]. To investigate if the antioxidants that inhibited ROS- and NET-production also affect other effector mechanism, we tested the effect of these antioxidants on neutrophil degranulation, chemotaxis, and phagocytosis (see supplementary materials and methods). DPI, an inhibitor of NADPH oxidase, was used to completely inhibit ROS production.

Neutrophil activation with 100 ng/mL LPS and 200 U/mL IFN*γ* or 20 nM PMA results in degranulation and in an enhanced cell surface expression of the granule membrane marker CD11b (Supplementary Figure 2). Neither treatment of cells with DPI nor with any of the tested antioxidants impaired activation-induced degranulation (Supplementary Figure 2).

The migration toward a chemotactic gradient in the presence of antioxidants was assessed in a transwell system. The used antioxidants as well as DPI had no inhibitory effect on neutrophil chemotaxis by using IL-8 (100 ng/mL) or TNF-*α* (100 ng/mL) as chemoattractant (Supplementary Figure 3).

The phagocytosis of fluorescent beads by neutrophils was examined by flow cytometry. Upon stimulation with 100 ng/mL LPS and 200 U/mL IFN*γ* and more stronger with 20 nM PMA, the phagocytic capacity of neutrophils increased (Supplementary Figure 4). While DPI significantly reduced the phagocytic capacity of neutrophils in response to LPS and IFN*γ* or PMA, the antioxidants used had no significant effect on neutrophil phagocytosis (Supplementary Figure 4).

## 4. Discussion

Although it has been reported that a few stimuli are able to induce a ROS-independent NET release [11–13], NETosis in response to most chemical and biological stimuli is mediated by ROS production involving NADPH oxidase and MPO [3, 4, 22, 45]. Since ROS-dependent NETosis is also believed to play detrimental effects in autoimmune inflammatory diseases, such as rheumatoid arthritis [16] or SLE [17], pharmacological inhibition of ROS-dependent NET formation could have a therapeutically effect on these disorders. Therefore, in the present comprehensive study we assessed the effect of a panel of substances with known antioxidative activity, such as flavonoids, vitamins, and pharmacological substances on the formation of ROS-dependent NETs by primary human neutrophils *in vitro*. We could convincingly show that all of the tested substances that significantly inhibited the production of ROS also inhibited the formation of NETs. Among these are the flavonoids epicatechin, catechin hydrate, and rutin trihydrate, vitamin C (ascorbic acid) and the substances 5-aminosalicylic acid and *N*-acetyl-L-cysteine. Other effector mechanisms, such as degranulation, chemotaxis, and phagocytosis, were not affected by these antioxidants.

The antioxidative effect of the tested substances is related to their ability to scavenge ROS like superoxide (O_2_^∙−^), hydroxyl radicals (OH^∙^), or hypochlorite (OCl^−^) and/or due to a direct inhibitory effect on ROS producing enzymes such as NADPH oxidase or MPO. Flavonoids have been described as scavengers for nitrogen, reactive oxygen, chlorine species, superoxide anion radicals, hydroxyl radicals, peroxyl radicals, hypochlorous acid, hydrogen peroxide [46–48], and inhibitors of MPO [24] and NADPH oxidase [49, 50]. The observed inhibitory effect of the flavonoids epicatechin and catechin hydrate on superoxide and/or MPO-dependent ROS can be related to their scavenging activity for OCl^−^ [48] as well as to their inhibitory effect on NADPH oxidase translocation and intracellular ROS production in neutrophils [50]. Furthermore, epicatechin can compete with hydrogen peroxide for compounds I and II of the MPO, resulting in decompensation of hydrogen peroxide during the epicatechin-driven peroxidation cycles of the MPO [39]. Therefore, the concentration-dependent significant inhibitory effect of epicatechin on MPO-specific ROS as measured by the luminol assay may be not only due to its scavenging activity but also due to its inhibitory activity on MPO. As MPO-dependent ROS and hydroxyl radicals as well as NADPH oxidase and MPO activity are essential for ROS dependent NETosis [3–9], we suggest that the flavonoids catechin hydrate and epicatechin inhibit NETosis through both their scavenging activity for OCl^−^ and regulatory function on ROS producing enzymes, especially on MPO. In an animal model for intestinal inflammation the antioxidative activity and ability to decrease neutrophil infiltration was recently confirmed in an *in vivo* system, indicating that the flavonoid epicatechin can be useful for preventing and treating intestinal inflammation [51].

Rutin trihydrate, a scavenger of superoxide [52], is the glycoside of the flavonoid quercetin, which has been shown to bind to a hydrophobic region at the distal heme pocket of the MPO leading to an inhibition of the MPO and decrease in MPO-dependent ROS [24]. For the binding and inhibitory effect of flavonoids on MPO, the C2 C3 double bound and the hydroxyl groups at the 3, 5, and 4′ positions are required. Rutin trihydrate fulfills these structural requirements except for the hydroxyl group at C3. In our study, rutin trihydrate, significantly inhibited the ROS-dependent NET formation. As we observed a dose-dependent antioxidative effect of rutin trihydrate on superoxide, as measured by the lucigenin assay, and on MPO-dependent ROS, as measured by the luminol assay [26, 30], one can speculate that the ROS- and NET-reducing effects of rutin trihydrate are not only due to its ability to scavenge superoxide [52] but also due to an inhibitory effect on the MPO.

Vitamin C (ascorbic acid) and vitamin E (*α*-tocopherol) are essential antioxidative micronutrients with a multiplicity of biological functions in humans. As scavengers of ROS, they have the ability to protect against oxidative damage to proteins, lipids, and nucleotides and were suggested to prevent cellular damage [53, 54]. Data concerning the effects of ascorbate on neutrophil ROS generation are controversial; both prooxidant [55] and antioxidant effects [56] have been described. Beside an antioxidative scavenging activity against hypochlorous acid [57, 58], ascorbic acid paradoxically has been reported to exert a stimulating effect on the chlorinating activity of the MPO, resulting in enhanced ROS production [58, 59]. The mode of action depends on the concentration of ascorbic acid. In low micromolar concentrations it has a catalytic stimulatory effect on MPO activity, while it acts as ROS scavenger in higher concentrations [58]. In our present study, where we used ascorbic acid in millimolar concentrations, we could observe a significant inhibitory effect of ascorbic acid on neutrophil ROS generation by using both the lucigenin- and the luminol-amplified chemiluminescence assay. Thus, our data indicate that ascorbic acid acts as scavenger against superoxide and HO^∙^ as well as MPO-dependent OCl^−^. Since these ROS are known to regulate NET release [4, 9], it is not surprising that the antioxidant scavenging activity of ascorbic acid leads to reduced NET release. A reduced NET formation by ascorbic acid was also recently described in response to monosodium urate [21]. Thus, vitamin C, in concentrations where it acts as ROS scavenger, may have a therapeutic effect on neutrophil/NET-associated diseases. This assumption is supported by a study in which adjuvant treatment of patients with ANCA-associated vasculitis with vitamin C led to reduced superoxide production by neutrophils [60]. As recently a direct inhibitory effect of ascorbic acid (in high millimolar concentrations of 50–200 mM) on MPO was shown in a cell-free system [61], we cannot exclude that inhibition of ROS and NETs by ascorbic acid in our experimental setting is only mediated by its scavenging activity.

Although vitamin E (*α*-tocopherol) is described as a chain-braking antioxidant that protects against lipid peroxidation through an inhibitory effect on protein kinase C (PKC) and NADPH oxidase as well as through its scavenging activity against peroxyl radicals [62], its antioxidant effect on neutrophils is controversial. While some studies reported an antioxidative impact of vitamin E on superoxide anion production by inhibition of PKC and NADPH oxidase in neutrophils [40, 56], no effect was observed on neutrophil-produced ROS in response to physiological stimuli [56]. In our present study we neither observe an anti-oxidative effect of *α*-tocopherol on PMA-stimulated ROS nor on NET release of human neutrophils. As vitamin E, in contrast to vitamin C, is unable to inhibit MPO activity [61], it is not surprising that we could not observe an inhibitory effect on MPO-dependent ROS as measured by the luminol assay. Since MPO activity and MPO-dependent ROS are essential for NETosis, it is not surprising that we observed no inhibitory effect on NETosis [4, 6, 45]. An explanation for the apparent lack of effect on superoxide production could be the synthetic *α*-tocopherol we used, which contains an equal mixture of eight different stereoisomers (RRR, RSR, RRS, RSS, SRR, SSR, SRS, SSS) [53]. Although all of them have antioxidant activities, only *α*-tocopherols in the 2R-configuration have strong biological activity and meet human vitamin E requirements [53]. Due to this, and since PMA used in this study is a strong synthetic inducer of ROS and NETs, the activity of the *α*-tocopherol used in this study could not be effective enough to inhibit the PMA-induced ROS and NETs.

5-Aminosaylicylic acid (5-ASA) is a cell permeable anti-inflammatory drug used in the treatment of inflammatory bowel diseases, such as ulcerative colitis and Crohn's disease [63]. These diseases are characterized by the extravasation and infiltration of large numbers of neutrophil granulocytes into the lamina propria, leading to injury and epithelial cell damage especially by synthesis and release of reactive oxygen species from neutrophils [64]. Although not yet clearly described, one can speculate that NETs may also play a role in the pathogenesis of inflammatory bowel diseases. This hypothesis is underlined by the demonstration of extracellular DNA in large bowel tissues from patients suffering from a bacterial gastrointestinal infection and Crohn's disease [65] and by activation of NET release in response to long pentraxin 3 (PTX3) [66, 67]. 5-ASA is an effective scavenger of neutrophil-derived ROS such as superoxide anions, hydroxyl radicals, singlet oxygen, amino chlorides, and especially hypochlorite [25, 36, 68, 69]. An effect of 5-ASA on MPO is discussed controversly. In a cell free system a direct inhibitory effect of 5-ASA on MPO has been described [70], while others found that inhibition of neutrophil generated ROS by 5-ASA is not associated with a decreased oxygen consumption *in vitro* [71], suggesting that the mode of action is the scavenging of ROS rather than enzyme inhibition. Thus, it is unclear whether 5-ASA, in addition to scavenge ROS, also inhibits ROS-producing enzymes *in vitro*. In most *in vitro* and *in vivo* studies the anti-inflammatory activity of 5-ASA is suggested to be related to its scavenging activity and not due to enzyme inhibition [69]. In our present study we observed an inhibitory effect of 5-ASA on superoxide and HO^∙^ as well as on MPO-derived OCl^−^ and for the first time on NET formation. Although we cannot exclude the possibility that a direct inhibitory effect on MPO contributes to the inhibition of ROS and NETs, we hypothesize in agreement with the literature, that the scavenging activity of 5-ASA mediates the anti-inflammatory effect on ROS and NETs [72]. Thus our results support the view regarding the therapeutic efficacy of 5-ASA in neutrophil-mediated diseases.

For the pharmacological substance *N*-acetylcysteine (NAC) we observed, in line with previous studies [9, 22], an inhibitory effect on PMA-induced ROS and NET production. These results, in addition to the observed anti-inflammatory effect of NAC on neutrophils from COPD patients [73], suggest a broad inhibitory role of NAC on ROS-mediated neutrophil functions. As a thiol-containing molecule NAC has well-documented antioxidant properties against ROS, such as H_2_O_2_ and HOCl *in vitro* and *in vivo* [57, 74–77]. Moreover, beside its direct antioxidant effect as free radical scavenger, NAC has an indirect antioxidant effect as prodrug of gluthathion that maintains/boosts the biosynthesis of reduced glutathione (GSH), the predominant antioxidant in the aqueous cytoplasm of cells [78]. A direct interaction of NAC with compounds I and II of MPO was shown *in vitro* [75, 79]. However, as in physiological conditions no formation of compound II could be observed [75] and the negatively net charge of NAC hampers the interaction with a negatively charged MPO [79] the theory supported the fact that thiols/NAC do not act by an inhibition of the MPO/H_2_O_2_/Cl^−^ system but by simple scavenging of ROS, especially HOCl [75, 79]. Since hypochlorous acids are important players in ROS-dependent NETosis [9], we suggest that NAC inhibits NET release through scavenging of these ROS.

Although a direct inhibitory effect of acetylsalicylic acid (ASS) in millimolar concentrations on neutrophil NADPH oxidase has been described [80], the antioxidant activity of ASS is suggested to be based on scavenging ROS but not on attenuating ROS generation [81]. As ASS is a potent scavenger of MPO-derived hydroxyl radicals/hypochlorous acid [81, 82] and does not significant react with superoxide [81] we expected an inhibitory effect on MPO-dependent ROS and NET production. Surprisingly we could not observe a significant inhibitory effect of ASS neither on ROS nor NET production of human neutrophils in concentrations up to 1 mM. These findings are in line with a recently published *in vitro* study on human neutrophils, in which an inhibitory effect of ASS on PMA- and TNF-*α*-induced NETs was observed only at a high concentration of 5 mM, but not for 1 mM [19]. Indeed, most studies analyzing the antioxidant activities of ASS used millimolar concentrations [80–82]. As we used PMA, a strong synthetic inducer of ROS and NET production, one can speculate that the antioxidative activity of ASS in concentrations up to 1 mM is not enough to exhibit an inhibitory effect. By using the physiological TLR ligand zymosan as stimulant, in a previous study an antioxidative effect of ASS on ROS production by human neutrophils was demonstrated [26].

The antioxidant effects of the hormone melatonin are related to its scavenging activity for ROS such as hydroxyl radicals [41, 83, 84] but also to a potent inhibitory effect on the catalytic activity of MPO [85]. An inhibitory effect of 2 mM melatonin on PMA stimulated intracellular ROS has been described for human neutrophils [41]. In our experimental setting we could observe a decrease of extracellular superoxide from melatonin treated neutrophils, but no inhibitory effect of melatonin on MPO-dependent ROS and NET formation. The lack of a decrease on MPO-dependent ROS could have various reasons. First, it was shown that melatonin dissolved in ethanol has less inhibitory effect on the luminol-detected luminescence than aqueous melatonin [86]. Furthermore, all recent studies regarding the effect of melatonin on MPO and/or ROS were carried out in different experimental settings, including different ROS detection methods and cell free systems [41, 86]. The inhibitory effect of melatonin on superoxide, but not MPO-dependent ROS in our experimental setting, seems not to be strong enough to inhibit the formation of NETs. These results support the findings that MPO and MPO-dependent intra- and extracellular ROS are crucial players of NETosis [4, 45].

Urea has no scavenging capacity and no influence on the ROS-mediated signal pathways. Therefore, it was used as a negative control. Hence, the lack of impact on the ROS formation and NET formation is not surprising.

## 5. Conclusions

In this study, we have shown that a broad spectrum of antioxidative substances that significantly inhibit the release of ROS by primary human neutrophils also inhibit the formation of ROS-dependent NETs, indicating a clear correlation between both phenomena. The inhibitory effect of the tested substances is related to their ability to scavenge ROS like superoxide, hydroxyl radicals, or hypochlorite and/or due to a direct inhibitory effect on ROS producing enzymes such as NADPH oxidase or MPO. For the flavonoids catechin hydrate, epicatechin, rutin trihydrate, and the pharmacological substance 5-ASA, we present evidence for the first time regarding their inhibitory effect on NET formation. Treatment with these substances may be considered as therapeutic strategies for neutrophil-mediates diseases, in which ROS and/or ROS-dependent NETosis play a role in pathogenesis.

## Supplementary Material

Figure S1. Antioxidants used in this study are not toxic and do not induce apoptosis in neutrophils.
Figure S2. Antioxidant treatment does not inhibit activation-induced degranulation of human neutrophils.
Figure S3. Antioxidant treatment does not inhibit chemotactic migration of human neutrophils.
Figure S4. DPI but not antioxidant treatment reduces phagocytic activity of stimulated human neutrophils.

## Abbreviations

OCl^−^: Hypochlorite
OH^∙^: Hydroxyl radicals
O_2_^∙−^: Superoxide
5-ASA: 5-Aminosalicylic acid
ASS: Acetylsalicylic acid
AUC: Area under the curve
DPI: Diphenyleniodiniumchlorid
DMSO: Dimethyl sulfoxide
EtOH: Ethanol
FM: Fluorescence microscopy
HOCl: Hypochlorous acid
Med.: Medium
MPO: Myeloperoxidase
NAC: *N*-acetyl-L-cysteine
NETs: Neutrophil extracellular traps
PMA: Phorbol myristate acetate
ROS: Reactive oxygen species
SEM: Scanning electron microscopy
Solv: Solvent.
