# Supplementary material for: Flavonoids and 5-Aminosalicylic Acid Inhibit the Formation of Neutrophil Extracellular Traps
Source: Mediators Inflamm. 2013 Dec 7;2013:710239. doi: 10.1155/2013/710239 (PMC3871909; doi:10.1155/2013/710239)
Supplement: Supplementary file 1 — Figure S1. Antioxidants used in this study are not toxic and do not induce apoptosis in neutrophils. Figure S2. Antioxidant treatment does not inhibit activation-induced degranulation of human neutrophils. Figure S3. Antioxidant treatment does not inhibit chemotactic migration of human neutrophils. Figure S4. DPI but not antioxidant treatment reduces phagocytic activity of stimulated human neutrophils. [file 710239.f1.pdf]

Supplementary Material and Methods

Analysis of neutrophil apoptosis and necrosis

Freshly isolated human neutrophils were centrifuged for 10 min, 1045 x g and suspended in NET medium (a custom-made modified RPMI-1640 medium without phenolred and sodium hydrogen carbonate containing 20 mM HEPES (Biochrom, Berlin, Germany), supplemented with 0.5 % human serum albumin (Behring, Marburg, Germany) and 10 mM HEPES buffer (PAA, Pasching, Austria)). Per sample, 0.5 x 10^6 neutrophils (5 x 10^6/ml) were seeded to a flat bottom transparent 96-well cell culture plate (Greiner-Bio-One, Solingen, Germany). After preincubation with or without the antioxidants for 5 h at 37 °C and 5 % CO_2, neutrophils were analyzed by staining with annexin V-FITC (Promokine, Heidelberg, Germany) and propidium iodide (Sigma-Aldrich). Apoptotic neutrophils flip the phosphatidylserine from the inner membrane to the outer membrane. Annexin -FITC binds to phosphatidylserine in a calcium-dependent manner whereas propidium iodide labels necrotic cells by intercalating into the DNA of dying neutrophils [1]. The double staining was performed as recommended by the manufacturers and neutrophils were analyzed immediately by flow cytometry using a FACS Calibur flow cytometer and the CellQuest Pro software (BD Biosciences, San Diego, USA).

Flow cytometry analysis of CD11b surface expression/degranulation

To examine the effect of the used antioxidants on neutrophil activation and degranulation the cell surface expression of CD11b was analyzed by flow cytometry. Following a 30 min pre-incubation with medium, solvent control (DMSO), various antioxidants or the NADPH oxidase inhibitor diphenyleniodiniumchlorid (DPI, 50 µM), 5 x 10^5 neutrophils in 100 µl were left unstimulated or activated either by 100 ng/ml LPS + 200 U/ml IFNγ or 20 nM PMA for 45 min at 37 °C, 5 % CO_2. After washing once with FACS-buffer (PBS pH 7.2 containing 1 % BSA (Roth, Karlsruhe,Germany), 1 % heat inactivated human serum and 0.01 % sodium azide (Sigma-Aldrich)) for 4 min at 300 x g, 4 °C, the cells were suspended in 100 µl FACS-buffer and stained with PE-conjugated mouse anti-human CD11b mAb (clone 2LPM19c, IgG1, DakoCytomation, Glostrup, Denmark) for 20 min on ice. PE-conjugated mouse IgG1 (BD Biosciences) were used as isotype controls. Finally, cells were washed twice with FACS buffer, fixed with 1 % paraformaldehyde (Sigma-Aldrich) and analyzed with FACS Calibur flow cytometer using CellQuest pro software.

Chemotaxis assay

The effect of antioxidants on neutrophil chemotaxis, was analyzed by a modified chemotaxis assay using a 24-well transwell system with 3 µm pore filters (Costar, Bodenheim, Germany) as previously described [2]. Briefly, 5 x 10^5 neutrophils (5 x 10^6/ml in complete medium without ß-mercaptoethanol) were pre-incubated for 30 min with medium, solvent (DMSO), an inhibitor of NADPH-oxidase (DPI) or various antioxidants in the upper chamber of the transwells. Then 100 ng/ml IL-8 (R&D systems, Abingdon, England) or 100 ng/ml TNF-α (PeproTech, Hamburg, Germany) in a volume of 600 µl were added in the lower chamber and the chambers were incubated for 30 min at 37 °C in a humidified atmosphere containing 5 % CO_2. The glucuronidase assay [3] was used to determine the number of cells that migrated toward IL-8 or TNF-α into the lower chamber. For this, cells that migrated to the lower chamber were lysed in 0.1 % TritonX-100 (Merck, Darmstadt, Germany) and glucuronidase enzymatic activity was measured using p-nitrophenyl-β-glucuronide (Sigma) as substrate. Finally the number of migrated cells was calculated with a standard curve that was obtained with lysates of known numbers of cells.

Phagocytosis assay

5 x 10^6 neutrophils/ml in complete medium were pre-incubated for 30 min with medium, an inhibitor of NADPH-oxidase (DPI), various antioxidants or solvent control (DMSO). Neutrophils were then left unstimulated or activated either with 100 ng/ml LPS and 200 U/ml IFNγ or 20 nM PMA and FluoSpheres carboxylate-modified microspheres with a diameter of 1 µM (Invitrogen) were added to a final concentration of 0.015 % (v/v) to the culture. After 30 min incubation at 37 °C and 5 % CO_2 non-phagocyted FluoSpheres were removed by washing the cells twice at 300 x g for 5 min at 4 °C. Cells were then suspended in 4 °C cold PBS containing 1% BSA (Roth) and 1 % heat inactivated human serum and were kept on ice. Subsequently phagocytosis of the yellow-green fluorescent FluoSpheres by neutrophils was assessed by flow cytometry.

References

1. Homburg, C.H., et al., *Human neutrophils lose their surface Fc gamma RIII and acquire Annexin V binding sites during apoptosis in vitro.* Blood, 1995. **85**(2): p. 532-40.
2. Wilde, I., et al., *Direct stimulatory effects of the TLR2/6 ligand bacterial lipopeptide MALP-2 on neutrophil granulocytes.* Med Microbiol Immunol, 2007. **196**(2): p. 61-71.
3. Ludwig, A., et al., *The CXC-chemokine neutrophil-activating peptide-2 induces two distinct optima of neutrophil chemotaxis by differential interaction with interleukin-8 receptors CXCR-1 and CXCR-2.* Blood, 1997. **90**(11): p. 4588-97.

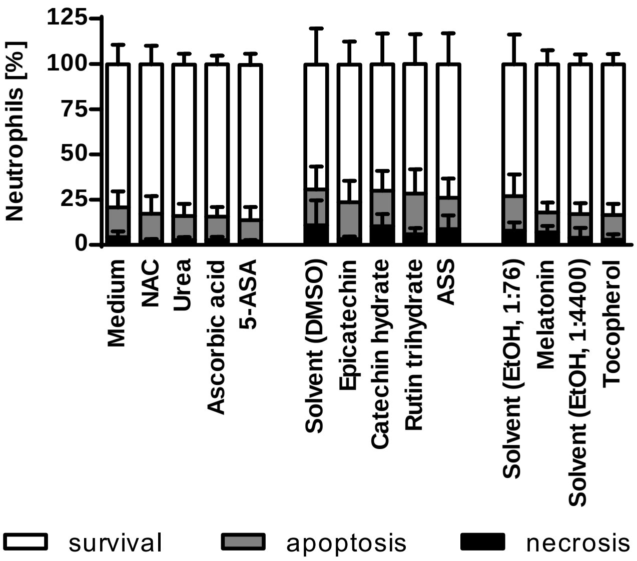

Figure S1 Antioxidants used in this study are not toxic and do not induce apoptosis in neutrophils.
Freshly isolated neutrophils were pre-incubated with or without various antioxidants (10 mM *N*-Acetyl-L-cysteine (NAC), 1 mM Urea, 2 mM ascorbic acid, 0.5 mM 5-aminosalicylic acid (5-ASA), 0.1 mM epicatechin, 0.1 mM catechin hydrate, 0.15 mM rutin trihydrate, 1 mM acetylsalicylic acid (ASS), 2 mM melatonin, 0.05 mM tocopherol) for 5 h at 37 °C. Apoptosis and cell death was analysed by flow cytometry using a staining with Annexin V-FITC and propidium iodide. DMSO was used as solvent control for epicatechin, catechin hydrate, rutin trihydrate and ASS and EtOH as solvent control for melatonin and tocopherol. Data show mean ± SD from 3 independent experiments.

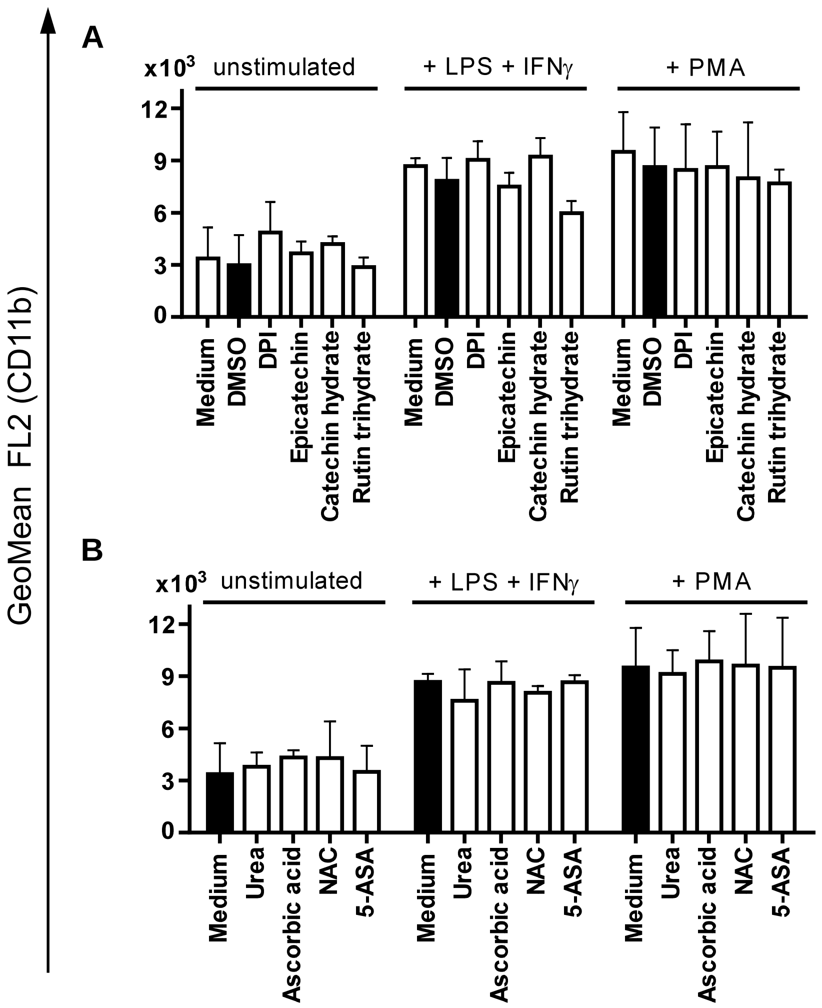

Figure S2 Antioxidant treatment does not inhibit activation-induced degranulation of human neutrophils.

Following a 30 min pre-incubation with medium, antioxidants or DMSO (solvent), 5 x 10^5 neutrophils in 100 µl were left unstimulated or activated either by 100 ng/ml LPS and 200 U/ml IFNγ or by 20 nM PMA for 45 min at 37°C, 5% CO_2. Surface expression of CD11b was assessed by flow cytometry after staining of the cells with PE-conjugated CD11b antibodies for neutrophils treatment with (A) 50 µM diphenyleniodiniumchlorid (DPI), 100 µM epicatechin, 100 µM catechin hydrate or 150 µM rutin trihydrate in comparison to solvent control (DMSO) and (B) 1 mM Urea, 2 mM ascorbic acid, 10 mM *N*-acetyl-L-cysteine (NAC) or 0.5 mM 5-aminosylicylic acid (5-ASA) in comparison to medium control. Data shown mean fluorescence intensities (geo mean FL2) ± SD from 3 independent experiments.

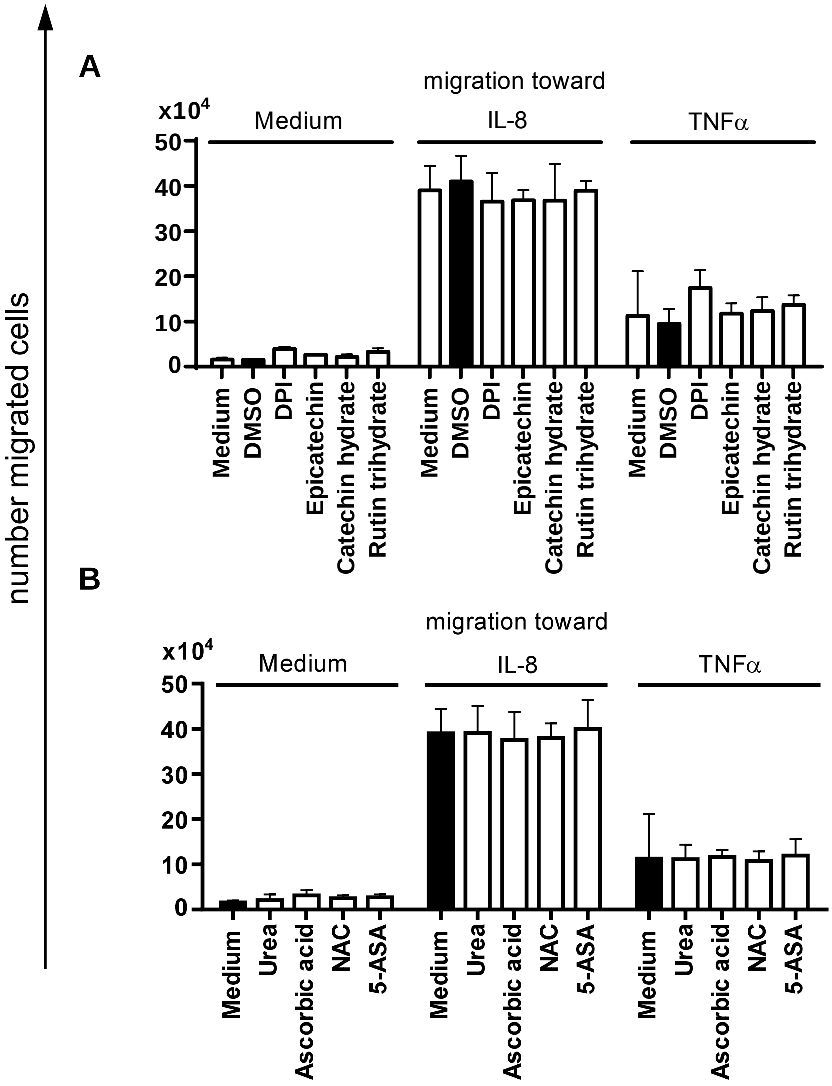

Figure S3 Antioxidant treatment does not inhibit chemotactic migration of human neutrophils.
Following a 30 min pre-incubation with medium, antioxidants or solvent control (DMSO), chemotactic migration of 5 x 10^5 neutrophils (5 x 10^6/ml) toward 100 ng/ml IL-8 or 100 ng/ml TNF-α was allowed for 30 min at 37 °C, 5 % CO_2 in the presence of (A) 50 µM diphenyleniodiniumchlorid (DPI), 100 µM epicatechin, 100 µM catechin hydrate or 150 µM rutin trihydrate in comparison to solvent control (DMSO) and (B) 1 mM Urea, 2 mM ascorbic acid, 10 mM *N*-acetyl-L-cysteine (NAC) or 0.5 mM 5-aminosalicylic acid (5-ASA) in comparison to medium control. Data show mean number of migrated cells ± SD from 3 independent experiments.

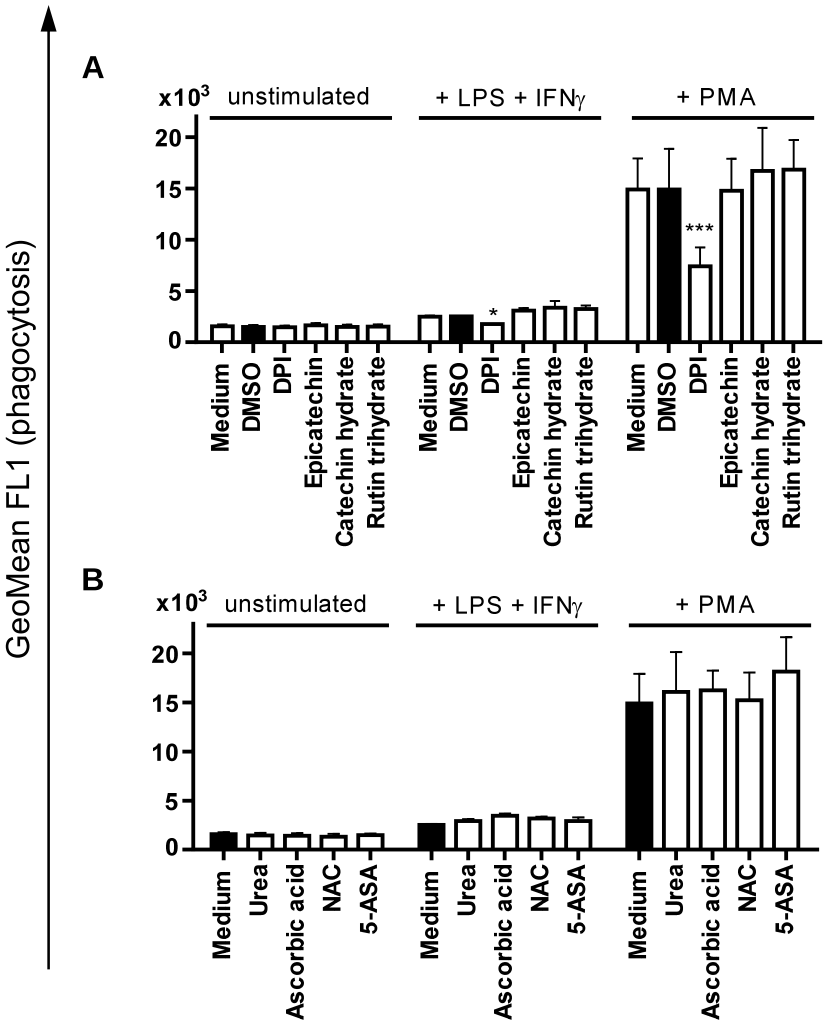

Fig. S4 DPI but not antioxidant treatment reduces phagocytic activity of stimulated human neutrophils.

Following a 30 min pre-incubation with medium, antioxidants or solvent control (DMSO), human neutrophils (5 x 10^6/ml) were co-incubated with FluoSpheres in the presence of medium, 100 ng/ml LPS and 200 U/ml IFNγ or 20 nM PMA for 30 min at 37°C, 5 % CO_2. Phagocytosis of fluorescent beads was then assessed by flow cytometry from neutrophils treated with (A) 50 µM diphenyleniodiniumchlorid (DPI), 100 µM epicatechin, 100 µM catechin hydrate or 150 µM rutin trihydrate in comparison to solvent control (DMSO) and (B) 1 mM Urea, 2 mM ascorbic acid, 10 mM *N*-acetyl-L-cysteine (NAC) or 0.5 mM 5-aminosalicylic acid (5-ASA) in comparison to medium control. Data show mean fluorescence intensities per cell (geo mean FL1) ± SD from 3 independent experiments. Significant differences were calculated with two way ANOVA followed by Dunnett`s method for multiple comparisons versus DMSO (A) or medium control (B). * $p < 0.05$ and *** $p < 0.001$.
